# Seeing is believing: Understanding the experiences and needs of marginalized communities living through successive disasters using photovoice

**DOI:** 10.1371/journal.pone.0337532

**Published:** 2025-11-26

**Authors:** Laura de la Roche, Carlos G. Fuentes, Ailiya Z. Jafry, Omolola E. Adepoju

**Affiliations:** 1 Humana Integrated Health System Sciences Institute, University of Houston, Houston, Texas, United States of America,; 2 Department of Health Systems and Population Health Sciences, University of Houston, Houston, Texas, United States of America; International University of Business Agriculture and Technology, BANGLADESH

## Abstract

**Introduction:**

Reports indicate increasing prevalence rates of successive natural disasters, and the negative impact on existing infrastructures are well documented. However, factors impacting outcomes on both communities and individuals remain unclear. For historically underserved communities, the effects of disasters are exacerbated by pre-existing barriers that make efforts to recovery difficult. Thus, understanding the nuance of their circumstances and experience is crucial to helping build resilience in these communities and inform preparedness and response efforts.

**Objective:**

Through this study, we sought to qualitatively understand the lived experience of historically underserved communities in the context of natural disasters to support the development and/or adoption of resources.

**Methods:**

Photovoice was used to guide semi-structured interviews with participants recruited from three communities (Kashmere Gardens, Greater Third Ward, Greater Fifth Ward) in Houston, TX. Reflexive thematic analysis was employed to generate themes accurately depicting participant experiences. Reflexivity, persistent observation, and triangulation were employed to increase trustworthiness in analysis.

**Results:**

Five key themes emerged from analysis: 1) Acute and long-term damage from natural disasters requires sustained recovery efforts; 2) Gaps and opportunities exist in city, state, and federal support mechanisms; 3) Strengthening and expanding support networks and community resources after extreme weather events is critical; 4) Need to address structural barriers to disaster preparedness and coping; and 5) Recognizing and mitigating the broad mental health impacts of natural disasters.

**Conclusion:**

The lived experiences of individuals from historically underserved communities in Houston highlight a complex interaction of psychosocial, structural, and cultural factors that influence both community resilience and vulnerability. Understanding this interplay is crucial to informing policy efforts that prioritize resilience building in these communities. Implications from these findings are discussed.

## Introduction

Over the last ten years, natural disasters in the United States have resulted in over 6,000 fatalities, costing over a trillion dollars in damage [1]. At an individual level, natural disasters are associated with reduced psychological and physical health and overall poor health outcomes [2–5]. Historically marginalized populations, such as older adults, patients living with substance abuse conditions, and people of lower socioeconomic status, are particularly vulnerable to negative outcomes during and following severe weather events [6]. Researchers have suggested that these populations are at greater risk of experiencing negative outcomes (e.g., poor psychological and physiological outcomes) due to limited access to resources and a lack of social support [6–8]. Addressing mental health care accessibility, housing availability and disaster preparedness is therefore necessary to support effective coping for these populations. Accordingly, there is a critical need to understand the lived experiences of marginalized communities in the context of natural disasters to support the development and/or adoption of resources, which requires qualitative investigations. Specifically, photovoice is a qualitative method that has been shown to be effective in understanding the factors that conceptualize the lived experiences of marginalized communities [9–11]. The research objective of this study is to assess the lived experiences of marginalized communities surviving successive disasters using photovoice.

The rates of mental health conditions (e.g., post-traumatic stress disorder (PTSD) and depression) are significantly elevated after a natural disaster [12–14]. Specifically, reports indicate nearly 1 in 5 individuals experiencing PTSD after major weather events, which is a significant increase from an average of 1 in 20 individuals directly following a natural disaster just a year earlier [12–14]. Individuals are often displaced after a natural disaster, and further report experiences of feeling isolated, encounters with financial exploitation or scams, and unsanitary living conditions [15]. Researchers have suggested that vulnerable populations such as women, the elderly, children, people of low socioeconomic status, or people with a past medical history of mental health challenges, are at greater risk of experiencing an increase in deleterious health effects after a natural disaster [16–18]. Coping with natural disasters has often incorporated culturally rooted strategies. For example, Sri Lankan adults relied on strategies established by tradition, culture, and religion to cope following a natural disaster [19] while those in Namibia relied on indigenous knowledge [20]. After Hurricane Katrina, interviews with survivors demonstrated the use of religious prayer, strength in spiritual beliefs, and faith in the community as a way to cope with the disaster and its negative impacts [21]. Similarly, following Hurricane Harvey, Vietnamese-Americans reporting finding strength in community [22]. Additionally, social support systems are reported to play a role in mitigating the adverse effects of natural disasters on mental health [23]. However, these are inaccessible to everyone, and many face barriers to developing strong social support systems [23,24]. The current literature underscores the need for targeted interventions.

The destructive impacts of natural disasters on infrastructure, society, and the economy are well documented through damaged houses, communities, hospitals and power grids, a loss of jobs resulting from damage to businesses, and a disruption of the water supply [25,26]. Immediately following a natural disaster, it is not uncommon for communities to be displaced due to a natural disaster [15]. Within these displaced communities, individuals report barriers to accessing clean water, adequate healthcare, food, and electricity, thereby increasing their risk of disease or injury [27]. Among historically marginalized communities, such as people of color, low socioeconomic households, or individuals with disabilities, recovery is significantly hindered by barriers and lack of support experienced pre-storm exacerbated by post-storm circumstances and is evident within multiple prior disasters [28]. For instance, in 2021, during the aftermath of Hurricane Ida, immigrant families in the US described living in basement apartments, sleeping on mattresses soaked in flood water with no access to heat for an entire month [29]. Similarly, after Hurricane Katrina, nearly half of the low-income parents studying in New Orleans were displaced, leading to increased housing instability [30]. Among parents, Black single mothers specifically experienced prolonged periods of multiple moves [31]. After Hurricane Harvey, low-income families experienced significant financial instability in part due to increased rental costs [32]. The immediate impacts of the storm exacerbated the effects of overcrowded living conditions.^26^ As time progresses, these impacts can prolong feelings of distress, which can perpetuate long-term mental health challenges, such as depression or PTSD [33].

Despite the need for targeted health resources to increase coping strategies and behaviors in these populations, research demonstrates a notable gap in the efficacy of existing disaster preparedness efforts and risk mitigation [34,35]. Successive disaster events continue to negatively impact communities, with re-occurring reports of negative psychological outcomes and housing crises and long-term negative outcomes, including housing availability and affordability [36]. The literature therefore highlights a gap in our knowledge of specific factors of lived experiences that are contributing to the negative outcomes reported, that may direct the development of targeted resources and disaster preparedness services and policies.

## Current study

The current study addresses the knowledge gap regarding specific factors that impact the psychological and physiological outcomes of individuals from underserved communities who survive successive disasters. Qualitative methods are necessary to obtaining rich and in-depth accounts of lived experiences to identify unknown influential factors [37,38], and accordingly, photovoice was employed. Specifically, a community-based participatory research method was utilized, in which individuals from communities submit and discuss photographs representing their experiences [35,39]. Photovoice has previously been utilized in various health settings, including identifying methods to improve family resilience in COVID-19, exploring themes of cultural competence within disaster relief workers, as well as assessing the sentiments of residents towards the prolonged impacts of natural disasters on their quality of life [37–39].This method amplifies the voices of marginalized communities, initiating pathways for collective action and can identify factors that are critical to consider in policy development [40].

## Methods

### Participants

In collaboration with community partners, participants were recruited from historically underserved communities in Houston Texas using community groups, online forums, and word-of-mouth. Participants were recruited from 09/01/2024 to 04/30/2025. Participants included 18 adults living in one and around three marginalized communities: Kashmere Gardens, Greater Third Ward, Greater Fifth Ward. These areas were selected due to the high proportion of minoritized residents, and the authors had a long history of working in these communities. The participants primarily identified as Hispanic (n = 8; 44% of participants) or African-American (n = 8; 44% of participants) and female (n = 17; 94% of participants); a portion of sample did not disclose their race/ethnicity (n = 2; 11%) and one participant identified as male (n = 1; 6% of participants).

### Procedure

Recruitment materials for the current study were distributed via virtual platforms, community discussions, and word-of-mouth. Specifically, recruitment details were posted on the virtual platforms used by non-profit organizations in the communities and paper copies of the flyer were distributed at community events and meetings. Participants who indicated they were interested in participating were contacted via email and/or phone call. Participants were eligible if they reported living in one of the three identified communities, were at least 18-years old, and verbal in English or Spanish. The study was explained to the participants and any questions answered; the link to the consent form on the Qualtrics platform was subsequently sent to the participant for review and contact information provided for any follow-up questions. Following consent receipt, follow-up emails were sent to establish an easy means for participants to submit their photos (via email). Participants were provided with a brief overview of photovoice and instructed to submit photos that represented their experience surviving natural disasters. Due to the nature of the topic of interest (i.e., the impact of natural disasters), we are not able to time data collection around a disaster event. Therefore, we allowed participants to submit new photos that represent their experience, or submit old photos they had taken when a previous natural disaster occurred in the past 5-years that represented their experience. Participants were asked to submit between two and five photos that would be later discussed during the individual interview. Following photo submission, a mutually convenient day and time was established for a virtual interview either via phone or zoom to discuss the photos submitted representing the participant’s experiences surviving a natural disaster. All interviews were audio-recorded and Zoom interviews were automatically transcribed using the Zoom transcription features. Ethics approval was obtained from the University of Houston Institutional Review Board – College of Medicine committee (IRB #00004432). Informed written consent was obtained from all participants.

### Measures

A semi-structured interview guide was developed for the individual interviews based on existing recommendations for photovoice studies. Specifically, the SHOWED method framework was utilized to develop the questions and guide the individual interview [41]. This method is a framework for organizing the discussion around submitted photos. The questions include: what to you **S**ee here?; What is really **H**appening here?; How does this relate to **O**ur lives?; **W**hy does this condition **E**xist?; What can we **D**o about it?. The SHOWED method targets the participants perception of their submitted photo and associated experience, and supports the introduction of a richer and more thorough exploration of what the photo and experience means and concerns or additional factors influencing that experience. Specifically, the interview guide asked participants how these photos and experiences related to their daily lives, what contributes to the perpetuation of the occurrences in the photo, and what could be done.

### Trustworthiness

Aligning with recommendations for ensuring rigor in qualitative research [38,42], multiple methods of trustworthiness were employed to increase the validity of our findings. Specifically, all authors engaged in reflexivity both individually, and through group discussions [43]; investigator triangulation was further incorporated to ensure diverse perspectives contributed to all components of the study, including data analysis. Further, negative case analysis was utilized to ensure all perceptions and positions regarding a topic were considered [44]. Finally, persistent observation of the data was conducted [45].

### Data analysis

All interviews were transcribed verbatim. Automated Zoom transcriptions were reviewed for accuracy and phone interviews were manually transcribed and double-checked by trained research assistants. Following transcription, the data was analyzed according to Braun and Clarke’s process and reporting guidelines for reflexive thematic analysis [46]. Specifically, this involved becoming familiar with the data, coding the transcribed interviews, generating and reviewing themes, and finally refining and organizing the generated themes and reporting the findings.

## Results

Participants submitted an average of 8 photos; a total of 142 photos were submitted across participants. The submitted photos varied in their content, and participants often discussed aspects of their experience that were not overtly evident in their submitted photos. Specifically, the submitted photos included damage to personal homes, infrastructure, and personal property. Additionally, some photos depicted experiencing surrounding travel, including flooded streets or modes of transportation (e.g., a friend’s car). Additionally, photos were submitted of the process of recovery and cleaning up following a disaster, including family and friends completing repairs, and debris within the community.

Analysis of the interviews generated five key themes regarding the lived experiences of participants from historically underserved communities who had survived successive natural disasters. De-identified quotes are included throughout the themes to exemplify participant voices as well as select photos that do not compromise participant confidentiality. Consent was obtained from participants to utilize de-identified verbatim quotes and photo submissions.

### Theme 1: Acute and long-term damage from natural disasters requires sustained recovery efforts

Participants described severe physical ailments that were attributed to extreme weather events and the environment they caused. That is, participants were not only injured as the natural disaster was actively occurring, but also following its completion in the damages the weather event caused. Participants reported high levels of exhaustion, which was not only attributed to the physical toll of the disaster, but also to the physical demands associated with having to individually manage housing and property damages. Furthermore, due to the community proximity to hazardous work sites and plants, the flooding was reported to introduce dangerous chemicals to individuals as they cleared debris and removed damage from their homes. Specifically, one participant stated that “the water is toxic” (CH008), while another described how exposure to flooding had made them “sick from the knees below, I got a rash, hives, an itch” (CH019). Physical ailments were often directly attributed to exposure to unclear water that contained dangerous chemicals and sewage.

**Fig 1:**
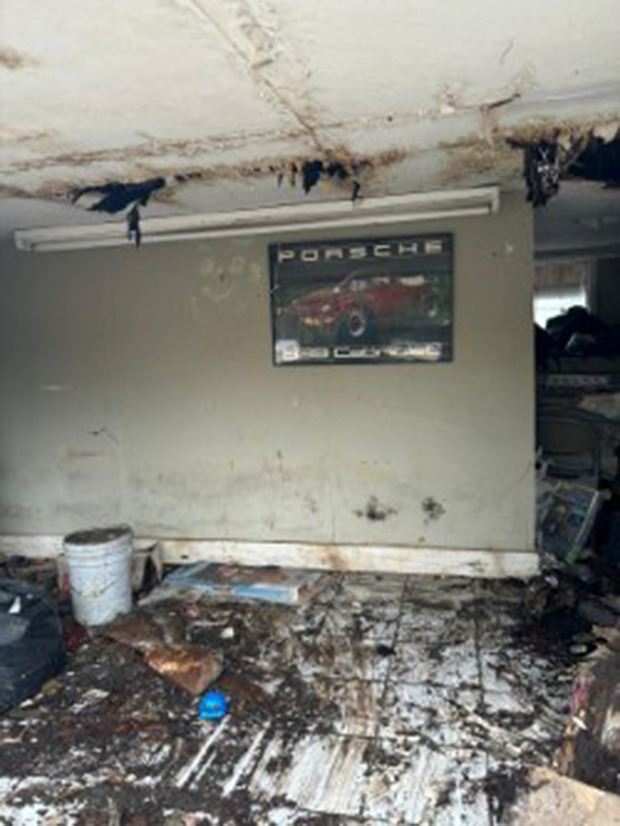
A submitted photo of the damage to a participant’s home following a hurricane.

Participants further identified severe damage to their property, their neighbors, and their community. Across different types of natural disaster events, ranging from severe storms to tornadoes and named hurricanes, participants described substantial damage to their residence and property that oftentimes made their home unsafe. The cause of the damage was often attributed to wind, trees and flooding, as one participant said “my ceiling came in on me during [Hurricane] Havey” (CH010). Pictures submitted of the impact of natural disasters were described as “basically just trees and destruction” (CH004). Notably, housing damage occurred to community residents even during storms that were not categorized as major weather events: “we have a lady, she’s got five kids, she has flooded five times. See, it doesn’t have to be a storm event or a rain or something like that, it can just be a heavy rain and we [the community] flood” (CH008) (Fig 1).

**Fig 2:**
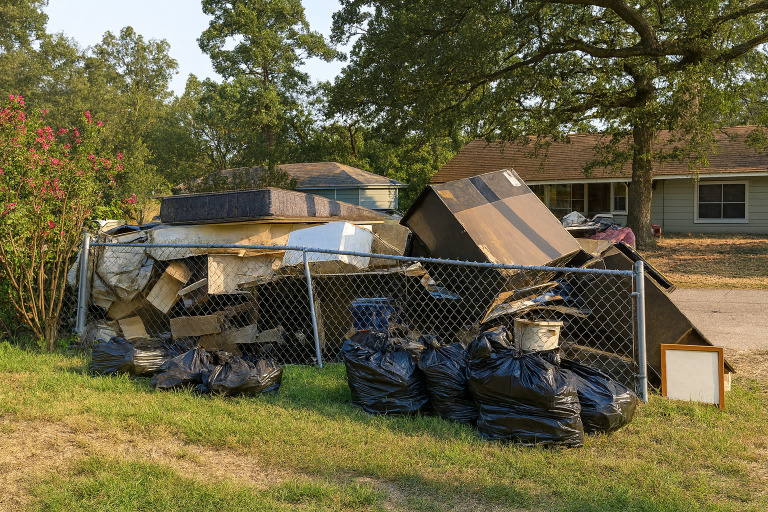
Submitted picture of built-up debris from homes in their neighborhood that were damaged following a hurricane, and had been sitting on the side of the road due to delayed city clean-up.

Damage was not only reported to individual homes, as one participant noted; “the tornado pretty much destroyed our community” (CH004). The location of industrial plants in close proximity to the community was further reported to negatively impact the entire community following major weather events. Specifically, one participant described how “[the recycling plant] has chemicals, when it rains and we have these disasters, these hurricanes, and it rains and floods, chemicals is floating back inside our homes” (CH018). While the negative health implications of the drainage from these plants have been identified by participants in the past and brough to the attention of city officials, participants felt that the “they care more about money than they do about the price of life” (CH015). Furthermore, damage and debris were reported to remain for extremely long periods of time (ranging from months to years) following a natural disaster due to the city not circulating throughout the community to pick it up: “I typically see the quality of the quality of the neighborhood going down. We still see tarps everywhere. We still see material that they have removed from their home on the side of the street. We still see ruined furniture” (CH015). Both acute damage occurring during a major weather event, as well as subsequent damage to the community following these events, were described (Fig 2).

### Theme 2: Gaps and opportunities exist in city, state, and federal support mechanisms

**Fig 3:**
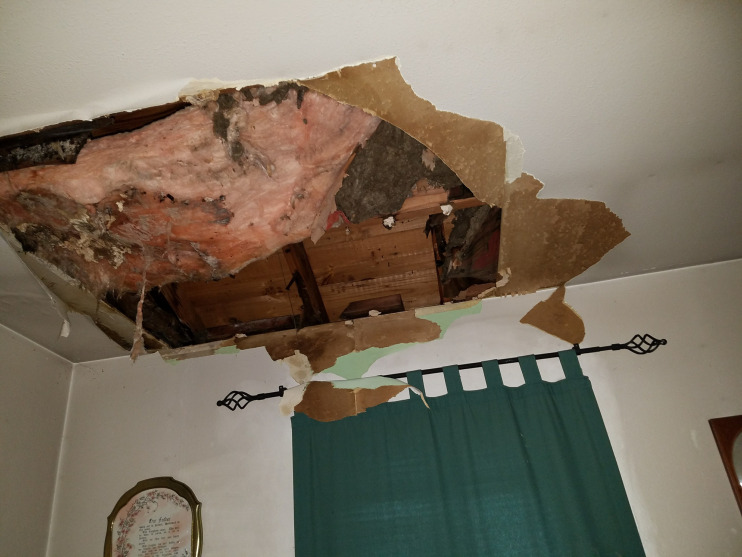
A participant described how despite the damage to their home, FEMA had denied their claim.

Following major weather events, participants voiced the immediate need for financial assistance due to the severity of the damage to both their homes and their community. Despite a clear need for financial assistance, very little federal or local support was reported. Specifically, while the Federal Emergency Management Agency (FEMA) was identified as a source financial aid, five participants articulated receiving minimal support from FEMA. Those participants indicated the funds they received did not come close to covering all of the damage and simple covered “miscellaneous things and food loss” (CH005). Conversely, over 50% of participants reported that FEMA was only known for denying community members without justification, as one participant stated “FEMA does not come to help us, FEMA comes to deny us” (CH008) and another described how “I spoke to FEMA and asked for help FEMA and they said no” (CH019) (Fig 3).

The language used in the FEMA applications was identified as confusing and purposefully deceiving, prompting members of the community to visit their local political representative to advocate for a formal review of the application forms and process. Despite a response that a review would take place and changes made, challenges associated with FEMA have reportedly continued. Participants felt the lack of attention to funding concerns was reflective of their community demographics (i.e., primarily African-American and Hispanic). Specifically, when calling the support line for receiving federal funding, a participant recalls how they “felt discriminated against because […] I am Mexican and I don’t speak English very well, I have an accent and everything […] but I try and communicate, and […] I don’t know what the problem is” (CH007). Perceptions of funding discrimination was further reported by CH019 who stated that “FEMA only help Blacks, they don’t help Hispanics […] they only look out for brown people, not for us”. This perceived funding discrimination extended beyond federal funding allocations and also included district and city funding. Specifically, participants reported being denied financial aid due to their zip code, as one participant recalls they applied for a city program and at “first [the city] said it wasn’t zip code based and [then] they said it was, so they kicked me out” (CH006). Funding allocated to the participants’ communities was further questioned, as participants noted that the responsibilities of the city, such as drainage, are not addressed in their community. Participants reported how they “found out that […] the money that […] should have been spent in that area [their community] went to another […] neighborhood that […] was higher [more affluent]” (CH010). The continued perceived discrimination in allocating funds to the participants’ communities resulted in legal action, in which

“we [the community] sued the state of Texas, General Land Office because they didn’t put any money back into the black and brown communities countywide. The money that the federal government had given them to disperse [..] they didn’t […] so we sued them under the title 6 [..] from the civil rights 1960 and 1964” (CH008).

Therefore, advocacy for fair funding distribution across communities was directed towards different levels of the government, including city, state, and federal.

### Theme 3: Strengthening and expanding support networks and community resources after extreme weather events is critical

Due to the lack of trust in receiving support from government agencies, participants relied on their communities, including family members, faither communities, and community organizations. For those who have family in the immediate area, they are often the first resource used following a major weather event. Community members and neighbors were further identified as instrumental support systems that share and spread knowledge and resources;

“the resources that [neighbor] knew of, she shared them with me, and […] then another friend of mine, and another friend of mine, and her friend, we pretty much just kind of network and we still are friends to this day […] we help our neighbors” (CH006).

**Fig 4:**
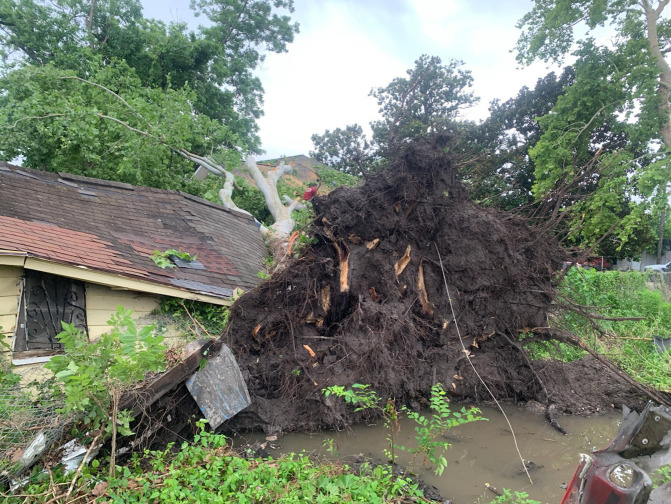
A participant submitted photo of a tree that had fallen on, and damaged their, and their neighbor’s property.

The importance of closely connected community members was highlighted by participants who reported going through disaster events alone in their home. Specifically, participants who were required to work, or had family members who had to work, were unable to be with family throughout a disaster. Therefore, additional support was often needed and provided via other community members (Fig 4).

Participants relied on their neighbors, describing how “we just try to pull together and help one another out you know, in hopes that […] our house will still be standing” (CH010). After the immediate danger of the weather event passed, this support translated to helping manage damages. Participants reported how they “saw neighbors helping […] neighbors helping out one another in the area […] taking out big amounts of sheetrock and trying to keep everything dry and putting tarps on homes’ (CH011).

Community support was reported to culminate in the formation of disaster support not-for-profits, as described by one participant:

“When it came time to evacuate out [of] here, one of those city trucks, no county, no nothing. Those young […] millennials, it as about 12 of them, they later formed West Street, they came with their boats and their kayaks and […] they started evacuating folks out [of] here. They came down [from] Boston, Maryland, Massachusetts, Niles, you know it was 12 friends that came and that was the beginning of West Street, Thank God” (CH008).

Participants reported relying on these organizations for accurate communication regarding incoming weather events. That is, they trusted these organizations to review and organize all available information on weather formations and developments and convey necessary information to them. Participants further described how they trusted these community organizations, such as West Street or NAC, due to the actions they had seen and the help and communication that had been provided. In fact, participants identified these groups as the emergency responders in their community following disasters: “believe it or not, they’re the first responders” (CH013). Furthermore, one participant described how they were just recently introduced to the West Street community organization felt this organization was doing a great job because “they call me – do you need this? Do you need water or ice or anything, tell us and then we can provide you with something that you need” (CH002).

Participants further discussed the formation and implementation of “hub houses” by these community groups. The hub house was described as having “all the things that you would need during, before, and after disasters, whether its medication, generators, certain types of medical equipment, PPE, stuff for cleaning up after the disaster” (CH005). Participants were often aware of the hub house that was closest to them, with one participant identifying that West Street currently had funding to support “between seven and ten, we’re trying to get funding possibly for more” (CH013). Across participants that were aware of hub houses, the most useful aspect they provided was typically back-up electricity via solar chargers or a generator. The provision of power was instrumental to participants to receive cold water and recharge necessary communication devices.

Across participants, there was significant and emotional gratitude expressed towards the existing community-led organizations (e.g., West Street) that could be relied upon to provide support and resources that were critical to surviving natural disaster events. When discussing these community organizations, participants likened them to family: “they [the community organization] are real supportive, we take care of each other, we are a family, we really don’t call ourselves a group, we call ourselves a big knit well blended family” (CH018).

In addition to organized non-profit community organizations, participants identified their faith communities as instrumental in providing pre- and post-disaster support. Prior to and during hurricane season, one participant described how their church gave out preparedness supplies to community members each month:

“The […] first Saturday of every month, [the church] have a drive-thru food pantry, fresh produce, some cleaning supplies, everything and they let you get up to four families as long as you have the individual’s ID, you know, to show and you register online […] and hey give out cleaning supplies - Sanitizers, gloves, personal hygiene, big cases of Frito-Lay, Chips, different kinds, […] household stuff. And when the hurricane hit, whenever there’s a disaster they’re going to have a real big giveaway. They give a lot. And so I tell my neighbors about that and I’ll pick up for them as well. They are good with giving mops, brooms, cleaning supplies, sanitizers, towels, soap, dishwashing liquid, bleach, everything” (CH006).

The provision of supplies from faith organizations was relied upon and perceived as trustworthy by community members. Specifically, faith centers were identified as safe locations that would provide shelter and aid during natural disaster events. Participants therefore reported that in future disasters they will “just go to the mosque who usually has […] generators and they […] serve as [a] disaster hub” (CH005). Faith organizations were further reported to provide supplemental funding for repairs following disaster events. Specifically, when participants were denied funding from insurance and federal programs, some reported finding funding support from faith organizations, including their local community church.

### Theme 4: Addressing structural barriers to disaster preparedness and coping

The level of preparedness and ability to do so varied across participants; while some participants reported they actively tried to prepare, others discussed an inability to do so. Specifically, while some described substantial preparedness efforts, they recognized the challenges associated with preparing and noted it had taken them time and saving to be able to obtain the different preparedness tools they needed. Participants reported working with their families and “learning together” (CH005) how to prepare for a major weather event. However, many participants feel unable to prepare due to compounding barriers, including financial constraints, time, and knowledge regarding how to prepare.

Those who could afford to do so reported upgrading their housing structures and installing generators or solar-powered electric options, as well as cutting down any trees surrounding their homes that could cause damage. For example, CH009 described how they “had the gentleman come out and he got rid of all the trees”. While eliminating risk from trees was identified as desired by participants, it often was simply not perceived to be feasible financially. A lack of perceived preparedness expressed by participants was therefore not by choice, but often due to a lack of feasibility. The struggle in preparedness efforts and feasibility to do so was further described by one participant who stated:

“then there’s really like procrastinating on doing it because I know that we don’t actually have the money for it. On preparing for disasters, […] on paying for climate mitigation and like adaptation ourselves […] most people just don’t have the money for it. And so I’ve been really procrastinating on […] even learning about all the things I should be doing because I’m like what will I do with that […] information” (CH005).

Participants reported seeking out information and leaning on communication and information provided by neighbors and non-profit community organizations in addition to individual research. Furthermore, participants discussed how recently, they found the news was providing them with more detailed information about incoming storms, allowing them more time to plan and prepare. However, participants reported a limited capacity to seek out information, especially when the suggestions were not perceived to be feasible. There was therefore a highlighted need by participants for education and resources on preparedness efforts and strategies that were feasible and affordable.

#### Necessary changes to support pre-disaster preparation and post-disaster coping.

**Fig 5:**
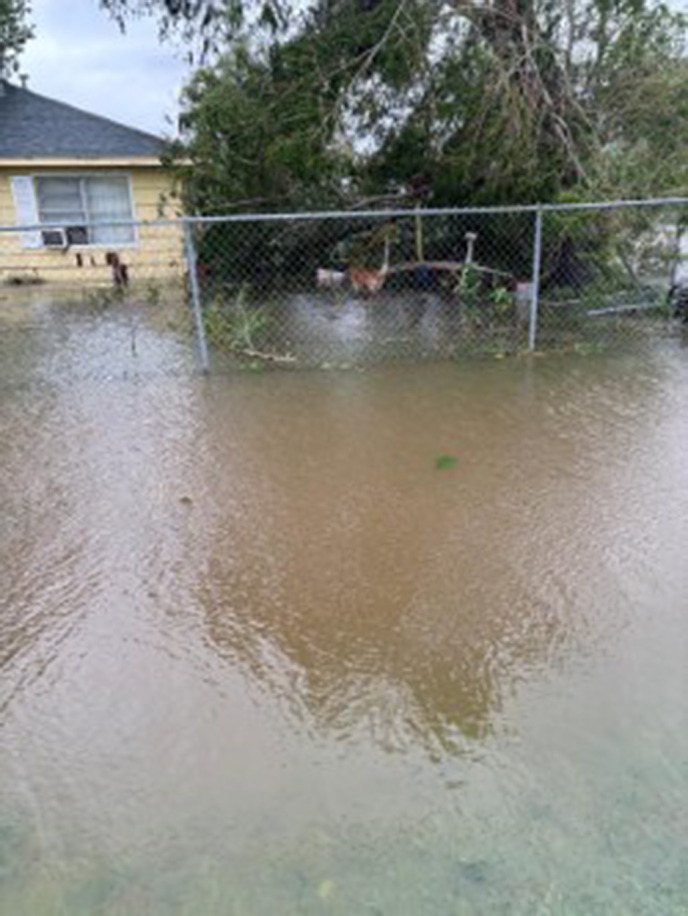
Submitted photo illustrating the severity of flooding in their community, highlighting problems with the existing drainage systems.

Discussions highlighted the positive impact that feeling prepared has on the psychological status of participants. That is, participants felt better about major weather events when they felt as though they had been able to sufficiently prepare, while not feeling prepared led to negative emotions: “I think that’s what a lot of people that I think that’s why a lot of people panic is because they’re not prepared” (CH006). To this end, they indicated that additional preparedness efforts to support the preparedness level of their community were needed. An identified aspect of preparedness efforts was targeting trees that could fall and cause damage to the community and the homes therein. That is, participants felt that the city should “cut the big branches [and] trim the trees” (CH002). Additional efforts by the city were identified as necessary by participants: “before the disaster, I feel that they could come through and clean the […] sewage drainage [and] clean the bayous […] somehow […] find a way that they can build a retention pond for us” (CH006) (Fig 5).

Additionally, to manage the acute psychological trauma of a natural disaster, followed by the subsequent negative psychological impact of post-disaster challenges (e.g., housing damage, insurance denial), participants discussed the critical need for the deployment of immediate psychological resources. Specifically, when asked about what resources they, their community, and their family need post-disaster, they identified available psychological professionals within their community to provide mental health support services for not only the trauma endured, but to educate them on coping strategies available to manage post-disaster environments. While there may be existing resources, they were not identified as accessible: “I just found out that Memorial Assistance Ministries has a free counseling program, but unfortunately […] there’s a super long wait list” (CH005). Participants highlighted the lack of communication regarding resources currently available in their community that were state or federally funded.

Participants identified additional novel resources they felt would be beneficial for their community, including a mobile psychological service following disasters. Specifically, they “wished [the city] had a van […] for mental health” (CH010). Non-medical psychological resources were also identified as beneficial and sought after. Specifically, participants perceived community group sessions to discuss their experiences to be extremely beneficial: “having group talks […] in the communities so we can help each other and better emerge victorious from this trauma, because not everyone has medical services where they can go” (CH007). Furthermore, participants felt that the service centers organized by the city were currently insufficient. Specifically, city-organized service centers providing a cool environment with survival necessities that opened in times of extreme weather events were not felt to be sufficient. Participants reported that these centers often did not have what they reported being able to provide, such as means to keep the center cool (i.e., generator), and often ran out of supplies (e.g., food, water). This resulted in community members attending these centers and not receiving any of the anticipated supplies, increasing the lack of trust felt by participants towards city initiatives. There was therefore a clearly articulated need for additional city-organized centers that were appropriately stocked with disaster necessities. Participants further recommended including more resources within these locations to adequately consider all needs of the community. One participant summed the suggestions up, describing that

“a church or something, the city could finance that, a resilience type hub where people can go and get information on how they can get financial help. They could have supplies at the at the resilience hub that people can either check out or they give. So they need a large location, really, in every community and especially the marginalized low income because we are the most vulnerable people” (CH004).

This participant did want to recognize some improvement in the city receiving suggestions from the historically underserved communities in Houston, explaining that “in fairness to the city, it seems as though they are trying to learn and work at how they can help the community, but it hasn’t come into fruition yet” (CH004). However, this was not voiced by most participants, who felt the city had not established a sufficient line of communication to hear the voices, concerns, and needs of the community. To that effect, many participants highlighted the importance of establishing open lines of communication between the community and city representatives. Specifically, across participants, communication was identified as the number one priority to ensure city awareness regarding community experiences and associated needs: “you have to, number one, be able to communicate with the people so you can know who needs help” (CH004). A communication line wherein participants feel heard and valued is not perceived to currently be in place by participants, who feel as though “we’re [the] sacrifice zones” (CH008). Participants therefore emphasized a need to establish a clear and efficient line of communication between community members and leaders and city representatives that can initiate positive change reflective of community needs.

### Theme 5: Recognizing and mitigating the broad mental health impacts of natural disasters.

The impact of natural disasters on participants’ psychological well-being was discussed across themes with participants reporting being “traumatized” (CH010). Importantly, the negative impact on mental health was not attributed to just one factor, but the cumulative effect of everything surrounding natural disaster ranging from preparation to coping with the fallout. Fear was a repeated emotion felt prior to and during a major weather event. The sense of vulnerability and helpless was reiterated, as participants described how they “were so afraid, but we couldn’t do anything” (CH002). During disaster events, such as hurricanes, participants reported turning to prayer as their primary coping mechanism, stating that “I pray, I pray” (CH016). One’s faith was identified as a critical mechanism on which to lean to decrease psychological distress in the midst of disaster events. Prayer was therefore relied upon through the entire cycle of disaster events, from when they are initially evident to their completion. One participant described how they coped as they watched the flood water rise around them: “I was like lord please. I don’t know how to swim, Its dark, please help me” (CH006). Participants often acknowledged prayer as their only coping method, not that “prayer, that’s all you can do” (CH013). Despite actively trying to cope with overwhelming emotions, fear of future major weather events was reported to negatively impact daily behaviors and intensify during any bad weather event, inclusive of smaller rain and/or thunderstorms.

Participants further reported rationalizing the event and associated overwhelming emotions, describing the experience as “you’re going on a survival journey and mentally it messes you up, but mentally you know you have to stay positive and focused that I’m surviving and this is something that I have to do” (CH006). Participants reported trying to adjust their perspectives to manage their emotional reaction to the devastation and damage from the disaster, describing how they “tried to go through the window of realizing that it’s bad for me, but it was worse for so many others. And so, I found myself looking at it and saying, Okay, I know this is bad, but I’m still blessed. I didn’t lose everything” (CH006). However, the ongoing damage that often remained for long periods of time following a natural disaster event reintroduced negative emotions, with participants noting that seeing the perpetuating damage “put[ting] me in a depression” (CH007). Some participants further identified themselves as leaders or role models within their community, and therefore felt they *couldn’t* experience poor mental health, identifying that while others report experiencing “PTSD” symptoms, they

“don’t have a chance to do that because I’m a leader in my community. So my adrenaline is like, are you okay? Are we okay? What can we do? How to help? So I don’t have a time to be depressed. It’s you go into survival mode for your community, for the people that you’ve been neighbors with for twenty plus years” (CH013).

Due to the negative psychological impact of natural disasters and navigating pre- and post-disaster environments, participants were concerned about others including other members of their community and their family: “I be worrying about my family” (CH016).

Participants further attributed the psychological impact of disasters on housing instability in their community, reporting that “instead of them [community members] getting help, it’s just hard for a lot of people to try to do what they have to do to get back up on their feet” (CH018). Negative mental health outcomes were further identified and related to all aspects surrounding natural disasters, inclusive of preparing for one and managing damages and processes to rebuild (e.g., obtaining funding) following the disaster event. Following major weather events, participants reported experiencing exhaustion, which was not only attributed to the physical demands of cleaning up damages and debris, but to the psychological toll of surviving through a disaster event. Additionally, participants discussed the impact managing finances and trying to receive financial aid and insurance compensation for disaster-related damages had on their mental health. Specifically, participants discussed the negative psychological impact they experienced receiving denials and no financial aid without perceived justification. This was highlighted by one participant who stated “I told [the insurance agent] you have made me suffer, I said, I don’t have to feel this way, [but] you are making me feel this way” (CH007).

Participants felt dismissed and ignored by companies and officials that they feel should be supporting them and their community in times of hardship, such as following a major weather event. Participants felt their mental health continued to be negatively impacted following natural disaster events, which they attributed to the remaining damage, issues with receiving insurance compensation or financial aid, and the lack of accountability and action taken by their city representatives. Specifically, one participant described how living through successive disasters “makes you angry, […] because this could be avoided, you’d be angry because here I am paying my tax money faithfully paying my bills faithfully and [the city is] not doing anything to protect us” (CH008).

## Discussion

The lived experiences of interview participants who have endured successive natural disasters in Houston reveal a complex interplay of psychosocial, structural, and cultural factors that shape community resilience and vulnerability. Five overarching themes emerged from the qualitative data: acute and long-term negative impacts, funding agencies, support networks and resources, feasibility of preparedness, and the impact on mental health. These themes not only underscore the multifaceted nature of disaster impact but also highlight critical gaps and strengths in individual and collective adaptive capacities. Framing these findings within the broader context of disaster resilience literature offers valuable insight into how urban populations – particularly in historically marginalized communities – navigate recurring environmental crises.

### Implications for preparing for, and recovering from, successive disasters

The photovoice evidence, including striking images of trees growing on rooftops and waist-deep floodwaters, provides a powerful illustration of the infrastructural and environmental challenges communities face during successive disasters. In discussions of disaster preparedness and the importance of communication, participants reported increased preparedness efforts by the city via news, mirroring other work examining news sharing and social media in efforts of disseminating disaster relief information, coordinating rescue and recovery activities [47], and ultimately promoting community resilience [48]. Community efforts remained a point of focus among participants, especially the establishments of self-sustained safe “hub” houses during disasters, and the organization of disaster preparedness kits and education for community members. These efforts, observed in many local organizations [49] are invaluable given that strong community-led preparedness initiatives have been proven to be more effective than government-led initiatives in developing community capacity [50]. These community programs ultimately have a protective effect, with work by Rivera et al. finding participation to be greater associated with increased odds of meeting preparedness indicators [51]. However, with participants also discussing individual-level preparedness efforts, concerns are raised as work suggests that low-income people are ultimately less likely to take disaster actions and are prepared for disasters than others [52]. These concerns are magnified by discussion by participants who stated they relied on prayer and did not engage in other preparedness efforts, a unique finding among disaster victims.

Both infrastructure and home damage resulting from disasters were identified across participants. Shared experiences with major flooding across houses and streets due to poorly maintained drainage systems align with previous work in similar communities, which have identified disadvantaged, minority-driven communities in Houston to be located closer to flood zones and more prone to home damage, and to also have ditches in disrepair with inadequate drainage [53]. Concerns regarding chemical run-off have also been reflected in work by Bodenreider et al., which found low-income, minority-driven communities to experience disproportionate exposure to chemical hazards mobilized by flooding [54]. Other housing-specific concerns, such as roof and structural damage from wind and long-term loss of power have also been noted in previous reports [55]. However, the contribution of these events to unrepaired, successive housing damage is a novel finding and stresses the dangers of infrastructural damage among a high-risk population that already struggles to rebuild and recover following disasters [56].

Frustrations regarding acquiring post-disaster financial support were shared among participants. Experiences and reviews from applying for FEMA assistance were mixed, with participants expressing difficulties in navigating the application process, deceitful jargon and questions, and either getting denied or receiving insufficient aid. Similar sentiments with FEMA have been shared in other work [57], particularly working with inexperienced agents and trying to file for aid online or at shelters following disaster. These frustrations lead to disparities in aid with findings by Raker and Woods showing that applications from poor, communities of color were disproportionately denied or delayed [58]. Increased homelessness following disasters due to inability to afford repair costs and alternate housing was also noted among participants, which aligns with findings which suggest low-income disaster victims to face the greatest difficulties rebuilding [50] and ultimately to face homelessness following disaster [59]. However, observations of lack of insurance coverage due to zip code are unique, echoing other redlining housing practices affecting the physical health of low-income communities [60]. As Houston is projected to continue experiencing successive natural disasters [61], it is crucial to take these findings and apply them in increasing community and individual-level preparedness efforts, and in advocating for infrastructure and city-level improvements to lessen the impact of these events on vulnerable populations.

From a policy angle, effective disaster risk management strategies might include community-based early warning systems, localized evacuation planning, and incentives for household-level mitigation measures. By linking community-generated evidence with institutional frameworks, multi-level strategies can be developed that integrate local knowledge into formal disaster preparedness and recovery policies, ensuring that interventions are both practical and grounded in the realities experienced by residents.

### Implications regarding the psychological impact and coping/support mechanisms

The participant narratives and images collected through photovoice highlight the significant psychological toll of successive disasters, revealing both stressors and coping strategies employed by community members. For instance, participants shared experiences of prolonged flooding and disrupted living conditions, which contributed to heightened anxiety and emotional distress. A broad range of emotional responses to disaster events were expressed among participants. Fear and stress expressed from the disaster and inability to stay with family due to work obligations have previously been expressed among disaster victims, where participants similarly expressed obsessive thinking, flashbacks, and fear every time it rained after a flood [57]. Moreover, feelings of resignation, the idea that ‘it is what it is’, has been captured in work by Osofsky et al., detailed as self-reliance, where adapting meant moving forward to many participants [62]. Additional feelings of gratitude from surviving disaster events is a reoccurring theme in disaster-related work, however pertaining more to survivor’s guilt among participants whose safety and homes remained mostly intact by disaster events [63]. However, concern continues to be raised as the inability to seek mental health services among participants aligns with findings that the majority of disaster survivors suffering from psychological symptoms [64] and low-income survivors [65] do not receive the mental health services they need. This inaccessibility in care is reflected in the linkage between trauma and stressors and mental health conditions such as depression and post-traumatic stress disorder (PTSD) following disaster events [66].

Community and family were noted as primary support structures among participants following a disaster. These findings regarding community support are congruent with other work covering Hurricane Harvey, which highlights the impact of various forms of organized mutual aid following the disaster [57]. With their mobile nature, these community organizations (e.g., churches, volunteer groups) are able to self-organize, leverage resources [67] and quickly provide on-the-ground, tangible support [68]. Moreover, community organizations are uniquely positioned with high credibility and trust among vulnerable populations needing the most help [69]. Moreover, findings of the word-of-mouth, family-like nature of these community networks are reflected in their ability to contribute to community disaster resilience [70]. Similar sentiments have also been shared in their fight to cover gaps in post-disaster management and advocacy for the community long after disaster events [71]. Ultimately, discussions around reliance on family as personal support systems, both physically and emotionally, have long been expressed in previous disaster-based literature as well [57].

City established Disaster Centers were characterized as underwhelming and under-resourced by participants, who stated centers to best serve as a provisionary source of food and safety, as they did not have air conditioning, stable power, nor were they always open despite being promised so. These grievances align with other work covering experiences in shelters, where disaster victims may have experienced magnified distress due to limited food and water [72], overcrowding [73] and inadequate medical and counseling resources [74]. The impact of these experiences are reflected in work by Kun et al., which found disaster victims residing in shelters or temporary homes to experience greater mental health risks like developing PTSD [75]; and are a reflection of empty city-level promises delivered to disaster victims in the aftermath of disasters. Investigations that focus on comparing the experiences and needs of individuals across different neighborhoods or precincts may provide further information on specific community needs.

The role of faith was greatly discussed among participants, particularly being identified as the primary, and only coping mechanism for many. Faith and religion have long been highlighted as coping strategies following major disasters [76]. The discussed ability of prayer to bring comfort during disaster events and ability to engage in post-disaster management aligns with the idea that faith-based congregations (FBCs) typically cover messages on overcoming adversity and grief during non-disaster times, which become critical tools following disasters [77]. Additionally, churches and other FBCs were praised for their quick turnaround in facilitating supplies and aid to communities, such as delivering essentials or aid in cleaning and gutting homes, which is in agreeance with work on FBCs during various natural disasters [78]. These experiences highlight the unique position of FBCs as community organizations that are both previously established and trusted [77], and can coordinate efforts with minimal downtime, making invaluable agents in coordinating disaster preparation and relief efforts.

Taken together, these findings underscore the need for policies that integrate mental health support into disaster preparedness and response programs, ensuring timely access to counseling, peer-support networks, and community-based coping resources. Institutional frameworks should prioritize psychosocial resilience by funding local mental health initiatives, training first responders in trauma-informed care, and incorporating community voices into program design.

## Limitations and future directions

This study is not without limitations. While participants were provided with different methods of contacting the research team and participating in this study, all those included ultimately chose to submit their photos via email and complete the interview either via zoom or audio call. This suggests that individuals uncomfortable with these technologies may have opted not to participate, and their perspectives are therefore absent from the findings. The inclusion of members from marginalized communities that are not comfortable with technology should be a priority in future investigations. It is also possible that there are nuanced differences exist across subgroups within these communities—such as by household income level or immigration status—that were not fully captured. Exploring these distinctions should be a priority for future investigations. Finally, we recognize that the use of photovoice centers primarily on the perspectives of community members, and the exclusion of institutional viewpoints from agencies such as FEMA, city management, and faith-based organizations can introduce bias. Future research should adopt a multi-stakeholder approach that integrates community voices with those of institutional actors to develop a more comprehensive understanding of disaster preparedness, response, and resilience planning.

Despite these limitations, this investigation obtained critical novel insight into the lived experiences of members of historically marginalized communities, and identified specific factors that impact their ability to prepare, respond, and recover from successive disasters.
